# CDK1, CCNB1, CDC20, BUB1, MAD2L1, MCM3, BUB1B, MCM2, and RFC4 May Be Potential Therapeutic Targets for Hepatocellular Carcinoma Using Integrated Bioinformatic Analysis

**DOI:** 10.1155/2019/1245072

**Published:** 2019-10-13

**Authors:** Wan-Xia Yang, Yun-Yan Pan, Chong-Ge You

**Affiliations:** Laboratory Medicine Center, Lanzhou University Second Hospital, Lanzhou, Gansu 730030, China

## Abstract

Hepatocellular carcinoma (HCC) is a malignant tumor with high mortality. The abnormal expression of genes is significantly related to the occurrence of HCC. The aim of this study was to explore the differentially expressed genes (DEGs) of HCC and to provide bioinformatics basis for the occurrence, prevention and treatment of HCC. The DEGs of HCC and normal tissues in GSE102079, GSE121248, GSE84402 and GSE60502 were obtained using R language. The GO function analysis and KEGG pathway enrichment analysis of DEGs were carried out using the DAVID database. Then, the protein–protein interaction (PPI) network was constructed using the STRING database. Hub genes were screened using Cytoscape software and verified using the GEPIA, UALCAN, and Oncomine database. We used HPA database to exhibit the differences in protein level of hub genes and used LinkedOmics to reveal the relationship between candidate genes and tumor clinical features. Finally, we obtained transcription factor (TF) of hub genes using NetworkAnalyst online tool. A total of 591 overlapping up-regulated genes were identified. These genes were related to cell cycle, DNA replication, pyrimidine metabolism, and p53 signaling pathway. Additionally, the GEPIA database showed that the CDK1, CCNB1, CDC20, BUB1, MAD2L1, MCM3, BUB1B, MCM2, and RFC4 were associated with the poor survival of HCC patients. UALCAN, Oncomine, and HPA databases and qRT-PCR confirmed that these genes were highly expressed in HCC tissues. LinkedOmics database indicated these genes were correlated with overall survival, pathologic stage, pathology T stage, race, and the age of onset. TF analysis showed that MYBL2, KDM5B, MYC, SOX2, and E2F4 were regulators to these nine hub genes. Overexpression of CDK1, CCNB1, CDC20, BUB1, MAD2L1, MCM3, BUB1B, MCM2, and RFC4 in tumor tissues predicted poor survival in HCC. They may be potential therapeutic targets for HCC.

## 1. Introduction

Currently, liver cancer is the fifth most common malignancy and the second most common cause of cancer death [1, 2]. Hepatocellular carcinoma (HCC) accounts for more than 90% of primary liver cancer, killing about 750,000 people worldwide each year [3]. In particular, the vast majority of cases (83%) have occurred in less developed regions of the world, causing a major health crisis in Asia [4]. Because 80–90% of liver cancer cannot be completely resected, and its' prognosis is very poor, it seriously threatens people's physical and mental health. According to the epidemiological investigation, it may be related to viral hepatitis [5], and drinking, moldy food, toxicants and genetic factors [6]. At present, more and more studies believe that the occurrence and poor prognosis of HCC are related to the abnormal expression of genes [7, 8]. However, since multiple genes are often involved in the process of cell carcinogenesis, and these genes can interact with each other and function through the regulatory network [9], the specific pathogenesis of HCC is still unclear. Although great efforts have been made to search for biomarkers of tumor prognosis or diagnosis, it is estimated that less than 1% of biomarkers are used in clinical practice [10]. Therefore, it is especially important to assess the relationship between biomarkers and diseases at the genetic level, protein levels and clinical factors.

In recent years, gene chip technology has played an important role in studying tumor gene expression profiles and searching for tumor key genes [11]. This study aims to provide bioinformatics basis for further research on the molecular mechanism of HCC, and provide a new way to carry out individualized treatment on genes level.

## 2. Materials and Methods

### 2.1. Microarray Data

In this study, the gene expression profile datasets (GSE102079, GSE121248, GSE84402, and GSE60502) were obtained from the Gene Expression Omnibus (GEO, https://www.ncbi.nlm.nih.gov/geo/) of NCBI. The microarray data from GSE102079, GSE121248, and GSE84402 were based on GPL570 (HG-U133_Plus_2) Affymetrix Human Genome U133 Plus 2.0 Array. The GSE102079 profile was composed of 152 HCC tissues and 91 nontumorous tissues. GSE121248 included 70 chronic hepatitis B-induced HCC tissues and 39 adjacent normal tissues. GSE84402 included 14 pairs of HCC tissues and corresponding nontumorous tissues. Totally, 18 pairs of HCC tissues and adjacent nontumorous tissues were enrolled in GSE60502 which was based on GPL96[HG-U133A] Affymetrix Human Genome U133A Array.

### 2.2. Screening Overlapping Up-Regulated DEGs in HCC

We used R software to analyze GSE102079, GSE121248, GSE84402, and GSE60502 raw data of the CEL file for identifying DEGs. RMA package was used for data normalization processing. Affy package was used for quality assessment of samples in each GEO dataset. The Limma package was used to identify DEGs. The criterion for selection of DEGs was set as |log_2_FC| > 1 and *P* value <0.05 for each GEO dataset. To identify shared up-regulated DEGs among GSE102079, GSE121248, GSE84402 and GSE60502, we used R software to generate a Venn diagram.

### 2.3. Function and Pathway Enrichment Analyses of Common Up-Regulated DEGs

To investigate the function of 591 common up-regulated DEGs, we used the Database for Annotation, Visualization and Integrated Discovery (DAVID, http://david.abcc.ncifcrf.gov/) online tool to perform functional and pathway enrichment analysis. Gene ontology (GO) analysis, including the biological process (BP), cellular component (CC), and molecular function (MF), and Kyoto Encyclopedia of Genes and Genomes (KEGG) pathway analysis were conducted for the selected common up-regulated DEGs using DAVID. *P* < 0.05 was regarded as statistical significance. Subsequently, the shared up-regulated DEGs enriched in top ten KEGG pathways/GO functions were determined for the Venn diagram using R software.

### 2.4. Protein–Protein Interaction (PPI) Network Construction and Hub Genes Selection

We used the Search Tool for the Retrieval of Interacting Genes (STRING, https://string-db.org/) online database to construct PPI network. CytoHubba was used to get the top 10 hub genes with the highest degree in the PPI network in Cytoscape software.

### 2.5. Validation of the Hub Genes

We used Gene Expression Profiling Interactive Analysis (GEPIA, http://gepia.cancer-pku.cn) to identify potential candidate biomarkers for overall survival (OS) and disease-free survival (DFS) in liver hepatocellular carcinoma (LIHC) patients. Genes that are significantly associated with OS and DFS were considered as potential biomarkers for LIHC prognosis. Furthermore, to evaluate mRNA expression of hub genes, we used UALCAN (http://ualcan.path.uab.edu/analysis.html) database and Oncomine (https://www.oncomine.org/) database to differentiate expression of hub genes in LIHC tissues and normal tissues.

### 2.6. Quantitative Real-Time PCR

HCC and adjacent tissues were taken from Cuiying center sample library of Lanzhou University Second Hospital. The total RNA was extracted by using TRNzol Reagent, and was reverse-transcribed with FastKing gDNA Dispelling RT SuperMix (TIANGEN, Beijing, China). All qRT-PCR reactions were conducted with Rotor-Gene 6000 PCR system (Qiagen) and performed with SsoFast EvaGreen Supermix (Bio-Rad) in 20 μl volume containing 10 μl of 2× SsoFast EvaGreen Supermix, 1 μl of each 10 μM forward and reverse primer, 1 μl of cDNA sample, and nuclease-free water up to 20 μl. Amplification was carried out according to the following conditions: initial denaturation 95°C 5 min, followed by 45 cycles of denaturation 95°C 10 s, annealing 57°C 15 s, extension 72°C 15 s. The relative expression of the gene was calculated by the 2^−ΔΔCt^method. The primers are listed in Table 1.

### 2.7. Evaluation of Immunohistochemical Staining

To verify the protein expression level of candidate genes in HCC tissues, we used Human Protein Atlas (HPA, https://www.proteinatlas.org/) database to obtain immunohistochemical staining.

### 2.8. Relationship between Candidate Genes and Clinical Features in HCC Patients

To further explore the relationship between candidate genes and tumor clinical features, we analyzed the TCGA clinical data using LinkedOmics (http://www.linkedomics.org/) database.

### 2.9. Transcription Factor (TF) and Expression Correlation Analyses

TF of hub genes was explored using NetworkAnalyst (http://www.networkanalyst.ca). Expression correlation analysis based on TCGA samples was conducted in GEPIA.

### 2.10. Statistical Analysis

Statistical analysis and graphs were performed with GraphPad Prism 7.00 sofware. Data were presented as the mean ± SD. The *t* test was used for comparison between the two groups.

## 3. Results

### 3.1. Screening Overlapping Up-Regulated DEGs

**Figure 1:**
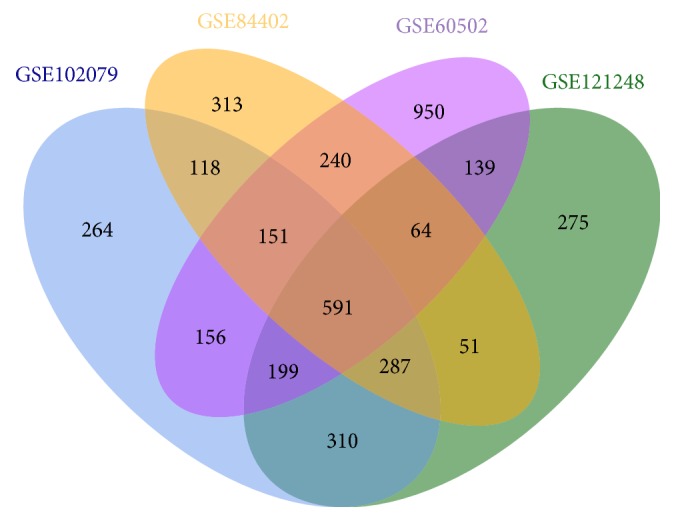
Identification of common up-regulated genes in gene expression datasets (GSE102079, GSE121248, GSE84402, and GSE60502).

We processed the data of four chips with R language, and set the cut-off criteria as |log_2_FC| > 1, the *p*-value <0.05 to screen the DEGs. A total of 591 overlapping up-regulated genes (2766 in GSE102079, 2483 in GSE121248, 2448 in GSE84402, and 3284 in GSE60502) were identified using a Venn diagram (Figure 1).

### 3.2. Functions and Pathways of Up-Regulated Genes

We presented the top ten pathways/GO functions in this study (Table 2). DEGs were mainly involved in biological processes such as cell division, sister chromatid cohesion, DNA replication, mitotic nuclear division, and G1/S transition of the mitotic cell cycle. Cytological composition analysis showed that most of these genes were involved in the composition of nucleoplasm, cytosol, nucleus, cytoplasm and membrane. The molecular functions were mainly concentrated in protein binding, poly (A) RNA binding, and ATP binding. KEGG pathway showed that the DEGs were mainly involved in the cell cycle, DNA replication, pyrimidine metabolism, and p53 signaling pathway.

**Figure 2:**
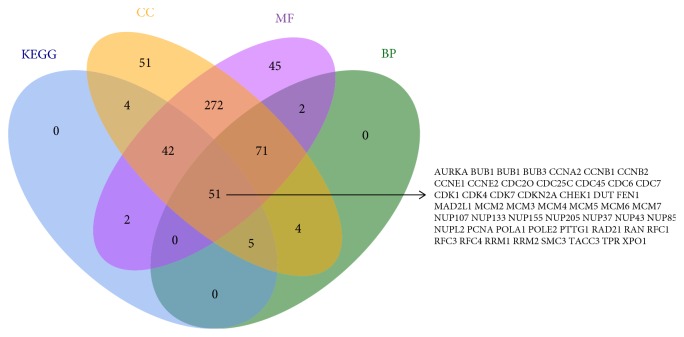
Overlapping up-regulated genes in KEGG, CC, BP and MF.

Additionally, 104 genes were enriched in KEGG pathways, 133 genes in biological processes, 500 genes in cellular component, and 485 genes in molecular function. Subsequently, we generated a Venn diagram for pathways/GO functions and obtained 51 overlapping genes. AURKA, BUB1, BUB1B, BUB3, CCNA2, CCNB1, CCNB2, CCNE1, CCNE2, CDC20, CDC25C, CDC45, CDC6, CDC7, CDK1, CDK4, CDK7, CDKN2A, CHEK1, DUT, FEN1, MAD2L1, MCM2, MCM3, MCM4, MCM5, MCM6, MCM7, NUP107, NUP133, NUP155, NUP205, NUP37, NUP43, NUP85, NUPL2, PCNA, POLA1,POLE2, PTTG1, RAD21, RAN, RFC1, RFC3, RFC4, RRM1, RRM2, SMC3, TACC3,TPR, and XPO1 were shared in the four datasets (Figure 2).

### 3.3. PPI Network Construction and Hub Genes Selection

**Figure 3:**
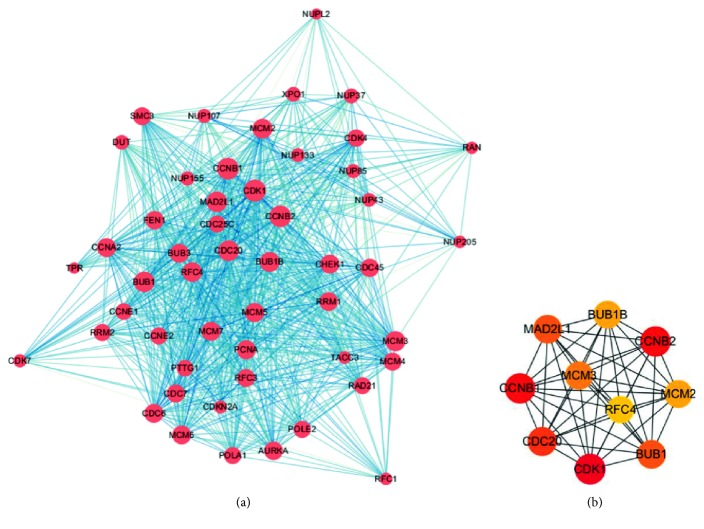
PPI network construction among DEGs and top 10 hub genes. (a) PPI relationships among DEGs. (b) Top 10 hub genes.

We constructed the PPI network for 51 overlapping up-regulated genes among KEGG pathway and GO analysis. The PPI included 51 nodes and 836 edges (Figure 3(a)). We used cytoHubba to get the top 10 hub genes with the highest degree of connectivity in the PPI network, and the top 10 hub genes included CDK1, CCNB2, CCNB1, CDC20, BUB1, MAD2L1, MCM3, BUB1B, MCM2, and RFC4 (Figure 3(b)). The connectivity degree of top 10 hub genes is shown in Table 3.

### 3.4. Survival Analysis of Top Ten Up-Regulated Genes

**Figure 4:**
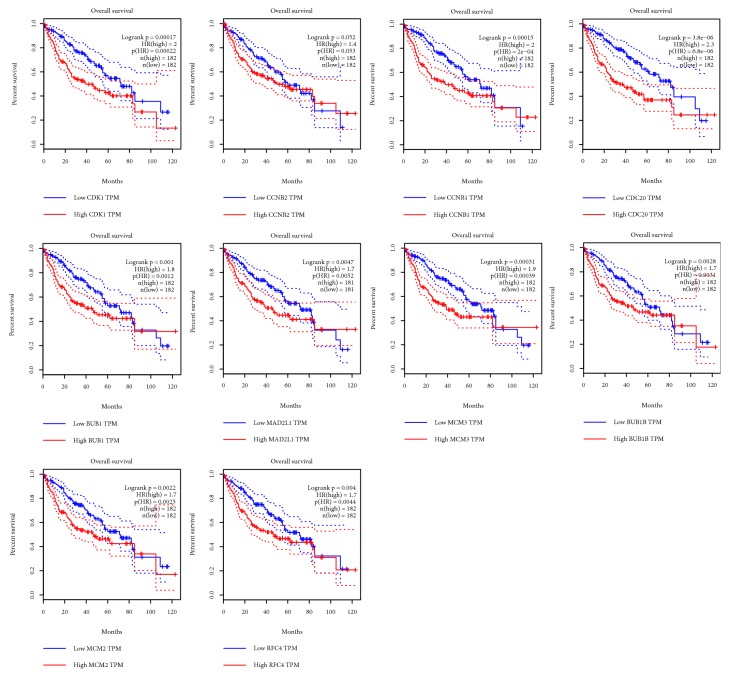
Overall survival validation of LIHC patients grouped by median cutoffs of CDK1, CCNB2, CCNB1, CDC20, BUB1, MAD2L1, MCM3, BUB1B, MCM2, and RFC4 in GEPIA.

**Figure 5:**
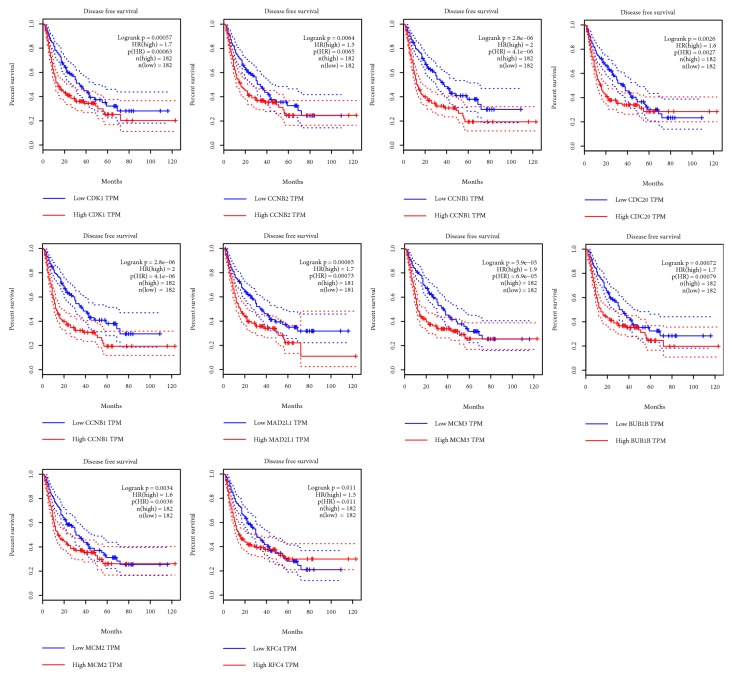
Disease-free survival validation of LIHC patients grouped by median cutoffs of CDK1, CCNB2, CCNB1, CDC20, BUB1, MAD2L1, MCM3, BUB1B, MCM2, and RFC4 in GEPIA.

We used GEPIA database to get the survival curves of 182 pairs of HCC tissues with high expression and low expression of hub genes. LIHC patients with high CDK1, CCNB1, CDC20, BUB1, MAD2L1, MCM3, BUB1B, MCM2, and RFC4 experienced poor OS; there was no statistical difference between CCNB2 expression and OS in LIHC patients (Figure 4). Similarly, compared with the low expression of these 10 genes, overexpression of CDK1, CCNB2, CCNB1, CDC20, BUB1, MAD2L1, MCM3, BUB1B, MCM2, and RFC4 in tumors was significantly associated with DFS in LIHC patients (Figure 5). Therefore, CDK1, CCNB1, CDC20, BUB1, MAD2L1, MCM3, BUB1B, MCM2, and RFC4 were considered as potential biomarkers.

### 3.5. Validation of Selected Up-Regulated Genes in HCC

**Figure 6:**
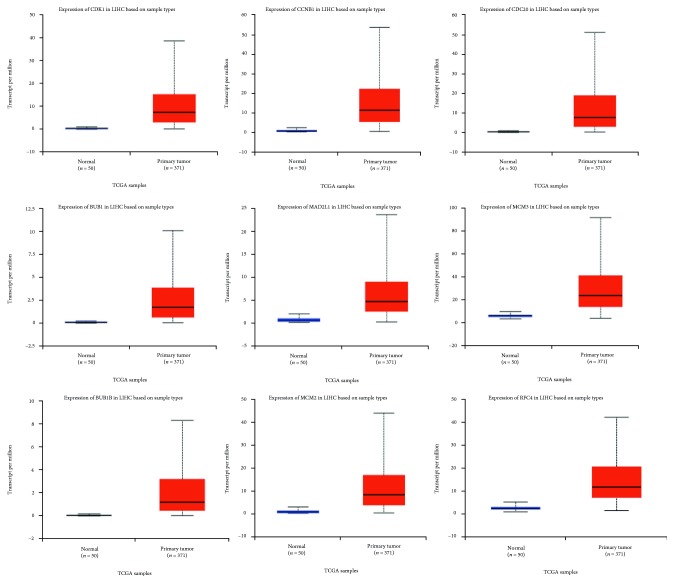
Validation of the expression of candidate genes in HCC tissues and normal tissues in UALCAN.

**Figure 7:**
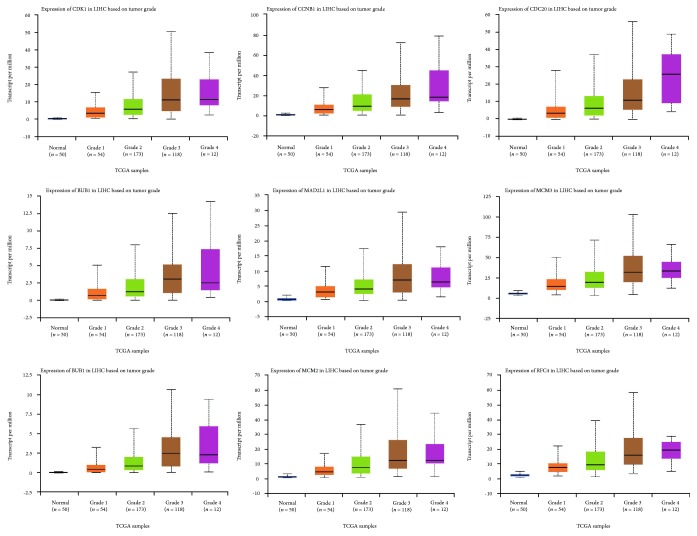
Validation of the expression of candidate genes in various grades of HCC tissues and normal tissues in UALCAN.

Using the TCGA data in UALCAN online tool, we analyzed the expression of the nine selected up-regulated genes in LIHC tissues (371 cases) and normal tissues (50 cases). The results showed that the CDK1, CCNB1, CDC20, BUB1, MAD2L1, MCM3, BUB1B, MCM2, and RFC4 were highly expressed in LIHC tissues, and the differences were statistically significant (Figure 6). Similarly, we also investigated the transcriptional levels of these genes in liver cancer and normal samples by using the Oncomine database. The mRNA expression levels of these genes were significantly up-regulated in liver cancer tissues compared with normal tissues in several datasets (Table 4). Furthermore, these 9 genes were also highly expressed in various grades of HCC compared with the normal group. In addition, overexpression of these 9 genes was also related to advanced tumor grade (Figure 7).

**Figure 8:**
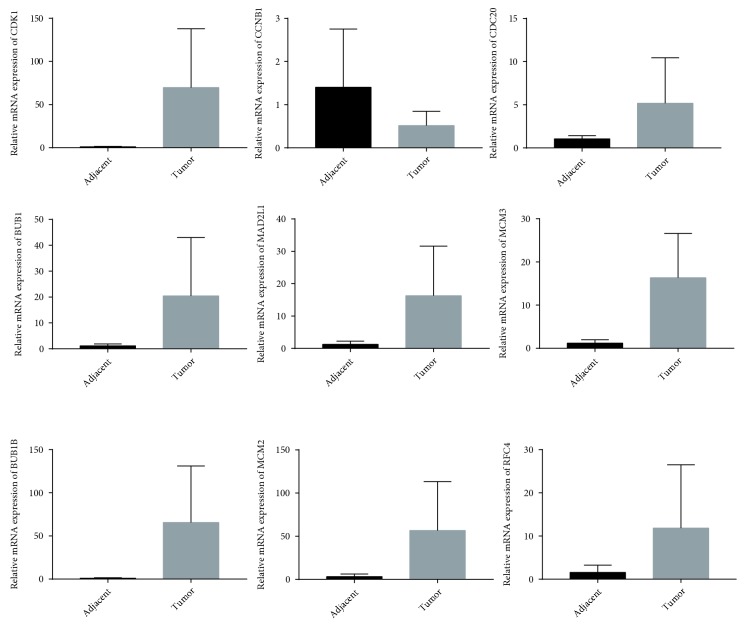
Relative mRNA expression of 9 candidate genes in HCC and adjacent tissues detected by qRT-PCR.

To further validate the data mining results, we performed qRT-PCR with paired tumor and adjacent tissues borrowed from the Cuiying center sample library of Lanzhou University Second Hospital. Although the sample was limited, except for CNB1, the other 8 candidate genes were highly expressed in tumor tissues (Figure 8).

### 3.6. Differences of Selected Up-Regulated Genes in Protein Level between HCC and Normal Tissues

**Figure 9:**
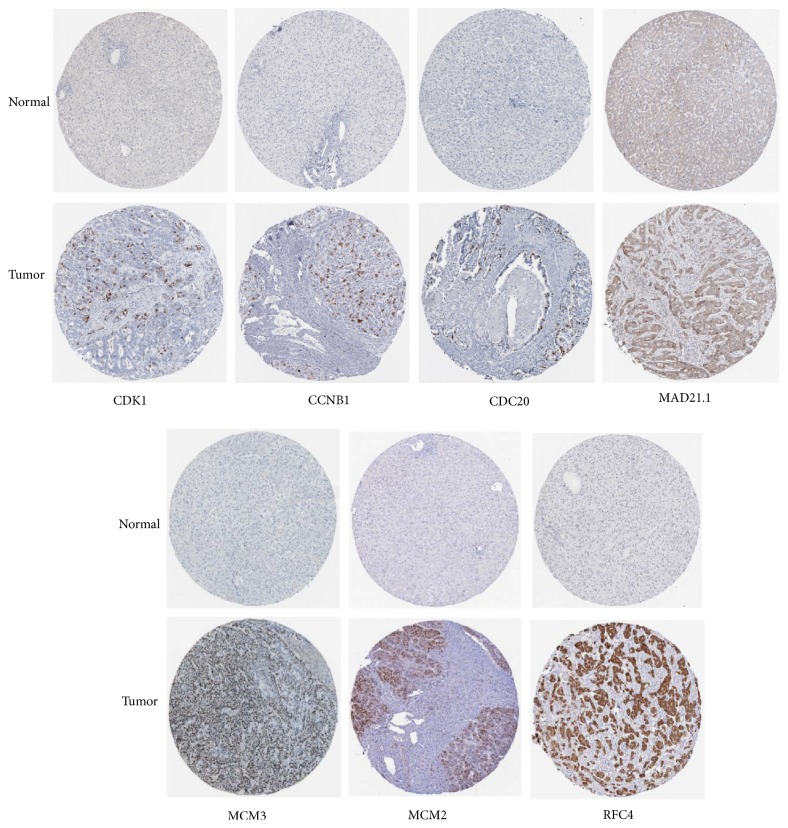
Immunohistochemical staining of candidate genes in HCC tissues and normal tissues in the HPA database.

We used the HPA database to exhibit the differences in protein level of CDK1, CCNB1, CDC20, MAD2L1, MCM3, MCM2, and RFC4. The results showed the immunohistochemical staining of CDK1, CCNB1, CDC20, MAD2L1, MCM3, MCM2, and RFC4 was negative staining in normal tissues and positive in HCC tissues, demonstrating that these genes were significantly expressed in HCC tissues than in normal liver tissues. The immunohistochemical staining is displayed in Figure 9.

### 3.7. Expression of Selected Up-Regulated Genes and Its Clinical Significance in LIHC Patients

Downloading the TCGA clinical data in LinkedOmics online tool, we analyzed the relationship between selected up-regulated genes and clinical features in LIHC patients. The results showed that all 9 candidate genes were significantly correlated with overall survival, pathologic stage, and pathology T stage, indicating that high expression of candidate genes predicted poor survival and tumor progression. The CDK1, CCNB1, CDC20, MCM3, BUB1B, MCM2, and RFC4 in LIHC patients were significantly correlated with race, which were significantly higher in Asian, Black, or African American and White than in American Indian or Alaska native, while BUB1, MAD2L1 were not significantly different in race. There was a significant correlation between CDK1, MCM3, BUB1B, RFC4 and the age of onset. The expression levels of the 9 genes in pathology N stage, pathology M stage, histological type, ethnicity, residual tumor, radiation therapy, and tumor purity were not statistically different (Table 5).

### 3.8. TF Analysis for Selected Up-Regulated Genes

**Figure 10:**
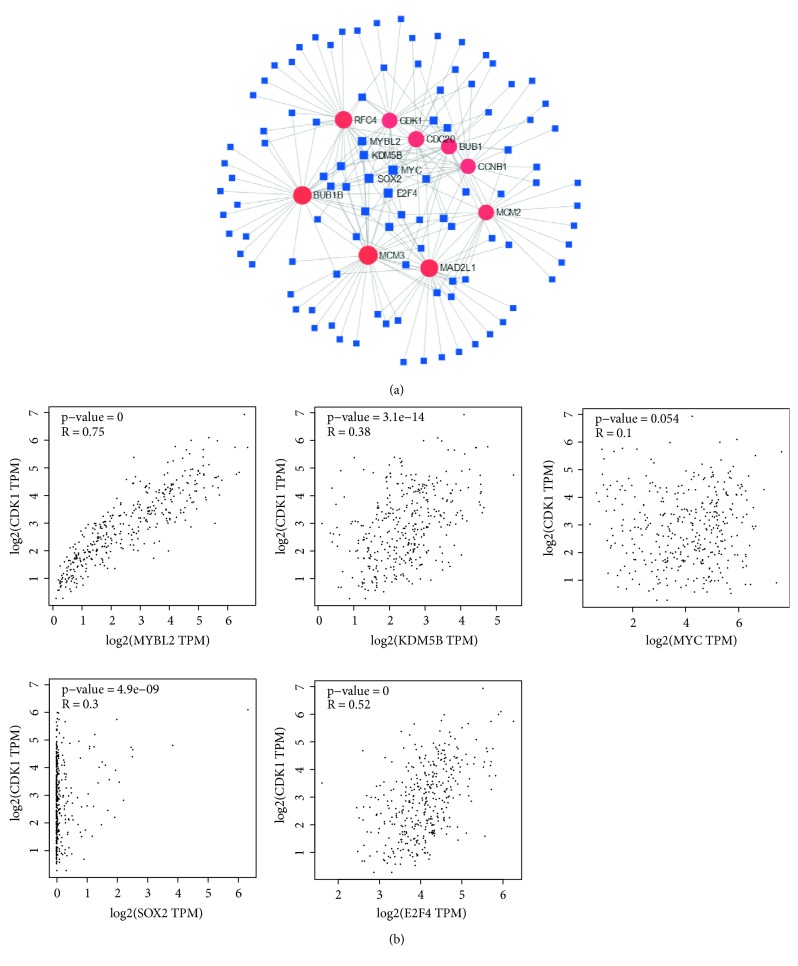
Association of TFs and selected up-regulated genes (CDK1, CCNB1, CDC20, BUB1, MAD2L1, MCM3, BUB1B, MCM2, and RFC4). (a) The network of TFs and selected up-regulated genes. (b) Correlation between TFs and CDK1 expressions.

We further investigated the molecular that can regulate CDK1, CCNB1, CDC20, BUB1, MAD2L1, MCM3, BUB1B, MCM2, and RFC4. We used NetworkAnalyst tool to predict the TFs that can regulate the expression of these nine genes. We found five TFs, MYBL2, KDM5B, MYC, SOX2, and E2F4, that can regulate these nine hub genes expression (Figure 10(a)). Correlation analysis showed MYBL2, KDM5B, SOX2, and E2F4 were positively correlated with these nine genes expressions. MYC was positively correlated with CCNB1, BUB1, MAD2L1, MCM3, and BUB1B expressions (Figure 10(b)).

## 4. Discussion

The occurrence of HCC is a complex biological process. In recent years, a large number of biomarkers have been used in the early diagnosis of HCC [15]. Many anti-HCC mechanisms have also been discovered [16]. However, there is still very little study at the multiple gene levels. The researches at the multi-gene levels can contribute to explore the pathogenesis of cancer. In this study, the data of four gene chips in HCC were analyzed by bioinformatics method. Finally, it was found that CDK1, CCNB1, CDC20, BUB1, MAD2L1, MCM3, BUB1B, MCM2, and RFC4 were related to the poor survival of HCC patients and the advanced tumor grade. A study had similar results [17]. The difference was that this study was validated at the transcriptional level and protein level. In addition, the study also analyzed the relationship between clinical features and biomarkers: these genes were correlated with overall survival, pathologic stage, pathology T stage, race and the age of onset. To explore the molecular mechanism of HCC, TF-hub genes regulatory network was also constructed. We identified 5 TFs, MYBL2, KDM5B, MYC, SOX2, and E2F4, all of which can regulate the expression of these 9 hub genes and provide more evidences for the elucidation of the mechanism of HCC progression.

Cyclin-dependent kinase 1 (CDK1) belongs to serine/threonine protein kinase family. A recent study has found that metformin can significantly inhibit the proliferation of HCC cells by inducing G2/M arrest and can effectively reduce the expression of CDK1 [18]. This result suggested that CDK1 may be involved in the process of cell proliferation in the cell cycle of HCC. Another study showed the miR-582-5p regulated the progression of HCC through directly inhibiting the expression of CDK1 and AKT3, and indirectly inhibiting the expression of cyclinD1 [19], and supported this theory. In addition, CDK1 is also expressed in other tumors. Studies have shown that CDK1 is active in the cell cycle of several tumor-regulating cell adhesion [20] and can be used as clinical prognostic biomarkers for nonsmall cell lung cancer [21], colon cancer [22], breast cancer [23], and ovarian cancer [24]. In this study, high expression of CCNB1 was closely associated with poor prognosis in HCC patients. This conclusion is further confirmed by a study that knockdown of CCNB1 regulated by microRNA-144 significantly inhibited cell proliferation, migration, and invasion in HCC [25]. In this study, the KEGG pathway showed that the DEGs were mainly involved in the cell cycle and DNA replication. Previous study [26] has shown that CCNB1/CDK1-mediated phosphorylation provides cells with efficient bioenergy for G2/M transition and shortens the overall cell-cycle time. Therefore, CCNB1/CDK1 plays an important role in the cell cycle and cell proliferation.

Overexpression of cell division cycle 20 (CDC20) is associated with poor prognosis of prostate cancer [27], breast cancer [28], and colon cancer [29]. However, the expression of CDC20 in HCC still lacks sufficient experimental data. In cutaneous squamous cell carcinoma, CDC20 promotes cell proliferation and migration through the Wnt/*β*-catenin signaling pathway [30]. CDC20 can contribute to cardiac hypertrophy by promoting LC3 degradation and inhibiting autophagy [31]. High expression of BUB1B can increase proliferation, migration, and invasion of prostate cancer cells [32]. MiR-200c-5p inhibits the proliferation, migration, and invasion of HCC cells by down-regulating MAD2L1 [33], which suggested that the expression of MAD2L1 is significantly higher in HCC and related to the poor prognosis of HCC. Moreover, it also indicated that MAD2L1 can be used as a prognostic and therapeutic target in HCC patients. The minichromosome maintenance (MCM) participates in DNA synthesis [34] and can be used as a biomarker of oral squamous cell carcinoma [35], melanoma [36], glioma [37] and colon cancer [38]. Replication factor C (RFC) plays an important role in DNA repair activities following DNA damage [39]. Targeted therapy of RFC3 can inhibit the proliferation and survival of HCC cells [40].

In this study, we identified DEGs in HCC by bioinformatics analysis and found that overexpression of CDK1, CCNB1, CDC20, BUB1, MAD2L1, MCM3, BUB1B, MCM2, and RFC4 in tumor tissues predicted poor survival in HCC. We hypothesized that CDK1, CCNB1, CDC20, BUB1, MAD2L1, MCM3, BUB1B, MCM2, and RFC4 may be potential therapeutic targets for HCC. We analyzed these genes at the transcriptional and protein levels, verified with qRT-PCR, explored the relationship between candidate genes and clinical factors, and constructed TF-hub genes regulatory network. Therefore, there are some advantages in this study.

## Figures and Tables

**Table 1:** List of primers.

Primer	Sequence
CDK1	Forward: 5′-CCCTTTAGCGCGGATCTACC-3′
	Reverse: 5′-CATGGCTACCACTTGACCTGT-3′
CCNB1	Forward: 5′-AATGGGAAGGGAGTGAGTGC-3′
	Reverse: 5′-GCATTAATTTTCGAGTTCCTGGTG-3′
CDC20	Forward: 5′-ATGCGCCAGAGGGTTATCAG-3′
	Reverse: 5′-AGGATGTCACCAGAGCTTGC-3′
BUB1	Forward: 5′-AGCCCAGACAGTAACAGACTC-3′
	Reverse: 5′-GTTGGCAACCTTATGTGTTTCAC-3′
MAD2L1	Forward: 5′-CGTGGCCGAGTTCTTCTCATT-3′
	Reverse: 5′-TACAAGCAAGGTGAGTCCGT-3′
MCM3	Forward: 5′-GCGACTTTGGTGGAGGTAGT-3′
	Reverse: 5′-TTGTTCAGAAGCCTCGTCGT-3′
BUB1B	Forward: 5′-TCTCAGAAACAGAATCCACGATCC-3′
	Reverse: 5′-TGCTAAATCTGCTATACCAAACAGG-3′
MCM2	Forward: 5′-GGTACTGCTATGGCGGAATCATC-3′
	Reverse: 5′-AAATGGTGGAAGGTCACGGC-3′
RFC4	Forward: 5′-AAGTCTCCTGGGCCCGTTAT-3′
	Reverse: 5′-CTTGCATGGTACTTCACCCAGT-3′
GAPDH	Forward: 5′-AGTGGCAAAGTGGAGATT-3′
	Reverse: 5′-GTGGAGTCATACTGGAACA-3′

**Table 2:** Enrichment analysis of GO and KEGG pathway of DEGs in HCC.

Category	ID	Term	Count	*P-*Value
BP	GO:0051301	Cell division	69	7.33E−33
BP	GO:0007062	Sister chromatid cohesion	32	1.74E−21
BP	GO:0006260	DNA replication	36	1.34E−19
BP	GO:0007067	Mitotic nuclear division	43	2.58E−18
BP	GO:0006270	DNA replication initiation	16	2.17E−14
BP	GO:0000082	G1/S transition of the mitotic cell cycle	24	2.84E−13
BP	GO:0007077	Mitotic nuclear envelope disassembly	15	9.00E−11
BP	GO:0031145	Anaphase-promoting complex-dependent catabolic process	18	8.02486E−10
BP	GO:0016925	Protein sumoylation	21	1.94799E−09
BP	GO:0000070	Mitotic sister chromatid segregation	11	3.5016E−09
CC	GO:0005654	Nucleoplasm	244	4.34338E−55
CC	GO:0005829	Cytosol	213	2.65555E−26
CC	GO:0005634	Nucleus	282	9.30863E−22
CC	GO:0005737	Cytoplasm	266	1.12033E−18
CC	GO:0016020	Membrane	143	3.98244E−17
CC	GO:0000777	Condensed chromosome kinetochore	25	2.062E−16
CC	GO:0000776	Kinetochore	24	4.34306E−16
CC	GO:0005635	Nuclear envelope	30	2.45558E−14
CC	GO:0005730	Nucleolus	68	1.1857E−11
CC	GO:0000775	Chromosome, centromeric region	17	1.72914E−11
MF	GO:0005515	Protein binding	452	1.69289E−45
MF	GO:0044822	poly(A) RNA binding	96	1.64645E−17
MF	GO:0005524	ATP binding	107	3.67235E−14
MF	GO:0003682	Chromatin binding	38	1.15215E−08
MF	GO:0019901	Protein kinase binding	37	1.32909E−08
MF	GO:0042393	Histone binding	15	5.49717E−05
MF	GO:0004674	Protein serine/threonine kinase activity	29	6.35606E−05
MF	GO:0003777	Microtubule motor activity	12	6.59963E−05
MF	GO:0051082	Unfolded protein binding	14	7.41926E−05
MF	GO:0003678	DNA helicase activity	7	0.000106899
KEGG pathway	hsa04110	Cell cycle	35	2.22E−19
KEGG pathway	hsa03030	DNA replication	15	6.16E−11
KEGG pathway	hsa00240	Pyrimidine metabolism	16	1.70E−05
KEGG pathway	hsa03013	RNA transport	21	2.97E−05
KEGG pathway	hsa04114	Oocyte meiosis	15	2.00E−04
KEGG pathway	hsa04115	p53 signaling pathway	11	4.22E−04
KEGG pathway	hsa03420	Nucleotide excision repair	9	6.48E−04
KEGG pathway	hsa03040	Spliceosome	15	0.001273995
KEGG pathway	hsa03430	Mismatch repair	6	0.002244294
KEGG pathway	hsa03022	Basal transcription factors	8	0.002420268

**Table 3:** Top 10 hub genes with the highest degree in the PPI network.

Gene symbol	Gene description	Degree
CDK1	Cyclin dependent kinase 1	49
CCNB2	Cyclin B2	48
CCNB1	Cyclin B1	48
CDC20	Cell division cycle 20	45
BUB1	BUB1 mitotic checkpoint serine/threonine kinase	44
MAD2L1	MAD2 mitotic arrest deficient-like 1 (yeast)	44
MCM3	Minichromosome maintenance complex component 3	43
BUB1B	BUB1 mitotic checkpoint serine/threonine kinase B	42
MCM2	Minichromosome maintenance complex component 2	42
RFC4	Replication factor C subunit 4	41

**Table 4:** Significant changes of candidate genes expression in transcription level in HCC.

Gene ID	Types of liver cancer versus normal	*p*-value	*t*-test	Fold change	References
CDK1	Hepatocellular carcinoma vs. normal	6.41E−29	13.87	4.148	Chen Liver [12]
	Hepatocellular carcinoma vs. normal	1.05E−84	28.109	5.573	Roessler Liver 2 [13]
	Hepatocellular carcinoma vs. normal	5.74E−10	7.891	8.68	Wurmbach Liver [14]
	Hepatocellular carcinoma vs. normal	9.36E−10	9.077	5.808	Roessler Liver [13]
CCNB1	Hepatocellular carcinoma vs. normal	6.06E−14	10.729	10.827	Wurmbach Liver [14]
	Hepatocellular carcinoma vs. normal	3.45E−88	30.468	5.783	Roessler Liver 2 [13]
	Hepatocellular carcinoma vs. normal	5.31E−08	7.428	3.901	Roessler Liver [13]
CDC20	Hepatocellular carcinoma vs. normal	5.17E−09	7.377	5.143	Wurmbach Liver [14]
	Hepatocellular carcinoma vs. normal	1.15E−64	22.985	3.814	Roessler Liver 2 [13]
	Hepatocellular carcinoma vs. normal	6.16E−08	7.171	3.661	Roessler Liver [13]
BUB1	Hepatocellular carcinoma vs. normal	1.57E−08	7.113	4.186	Wurmbach Liver [14]
	Hepatocellular carcinoma vs. normal	2.37E−15	8.643	2.808	Chen Liver [12]
	Hepatocellular carcinoma vs. normal	5.27E−07	6.531	2.046	Roessler Liver [13]
	Hepatocellular Carcinoma vs. Normal	1.35E−46	17.257	2	Roessler Liver 2 [13]
MAD2L1	Hepatocellular carcinoma vs. normal	6.77E−20	10.263	2.43	Chen Liver [12]
	Hepatocellular carcinoma vs. normal	3.63E−56	20.301	2.884	Roessler Liver 2 [13]
	Hepatocellular carcinoma vs. normal	2.40E−07	6.715	2.548	Roessler Liver [13]
	Hepatocellular carcinoma vs. normal	3.52E−06	5.131	3.669	Wurmbach Liver [14]
MCM3	Hepatocellular carcinoma vs. normal	5.67E−72	23.777	3.023	Roessler Liver 2 [13]
	Hepatocellular carcinoma vs. normal	1.45E−08	7.55	2.953	Roessler Liver [13]
BUB1B	Hepatocellular Carcinoma vs. Normal	1.29E−11	8.907	6.747	Wurmbach Liver [14]
	Hepatocellular carcinoma vs. normal	4.52E−10	9.2	4.407	Roessler Liver [13]
	Hepatocellular carcinoma vs. normal	4.23E−64	22.98	3.34	Roessler Liver 2 [13]
	Hepatocellular Carcinoma vs. Normal	1.94E−08	5.795	2.55	Chen Liver [12]
MCM2	Hepatocellular carcinoma vs. normal	2.68E−64	21.853	3.144	Roessler Liver 2 [13]
	Hepatocellular Carcinoma vs. Normal	3.00E−08	7.289	3.252	Roessler Liver [13]
RFC4	Hepatocellular carcinoma vs. normal	1.15E−88	28.334	4.6	Roessler Liver 2 [13]
	Hepatocellular carcinoma vs. normal	5.28E−19	9.909	2.031	Chen Liver [12]
	Hepatocellular carcinoma vs. normal	4.58E−08	7.428	3.315	Roessler Liver [13]

**Table 5:** Relationship between selected up-regulated genes and clinical features in HCC.

		CDK1		CCNB1		CDC20		B UB1		MAD2L1		MCM3		BUB1B		MCM2		RFC4	
Item	*N*	Statistic	*P*-value	Statistic	*P*-value	Statistic	*P*-value	Statistic	*P*-value	Statistic	*P*-value	Statistic	*P*-value	Statistic	*P*-value	Statistic	*P*-value	Statistic	*P*-value
years_to_birth (Spearman's correlation)	340	−0.16	0.00	−0.09	0.09	−0.09	0.11	−0.09	0.09	−0.04	0.43	−0.11	0.04	−0.12	0.03	−0.10	0.07	−0.12	0.02
Tumor_purity (Spearma's correlation)	343	−0.01	0.82	−0.02	0.70	−0.06	0.24	−0.05	0.32	−0.09	0.08	0.09	0.10	−0.01	0.85	0.02	0.76	0.00	0.99
overall_survival (Cox regression test)	alive 220	0.26	0.00	0.34	0.00	0.29	0.00	0.25	0.00	0.34	0.00	0.32	0.00	0.24	0.00	0.31	0.00	0.37	0.00
	dead 123																		
pathologic_stage (Kruskal-Wallis test)	stage I 161	19.14	0.00	21.37	0.00	19.04	0.00	23.47	0.00	23.03	0.00	16.08	0.00	22.74	0.00	13.83	0.00	14.63	0.00
	stage II 77																		
	stage III 80																		
	stage IV 3																		
race (Kruskal-Wallis test)	american Indian or alaska native 1	13.12	0.00	15.19	0.00	14.12	0.00	7.51	0.06	1.05	0.79	20.12	0.00	10.93	0.01	17.21	0.00	21.99	0.00
	asian 148																		
	Black or arican american 15																		
	white 169																		
pathology_T_stage (Kruskal-Wallis test)	t1 168	17.25	0.00	20.33	0.00	19.96	0.00	23.64	0.00	24.03	0.00	14.16	0.00	19.89	0.00	12.42	0.01	11.72	0.01
	t2 84																		
	t3 75																		
	t4 13																		
pathology_N_stage (Wilcox test)	n0 239	0.09	0.20	−0.01	0.91	0.15	0.67	0.09	0.28	0.20	0.09	0.03	0.35	0.17	0.18	0.14	0.58	0.11	0.26
	n1 3																		
pathology_M_stage (Wilcox test)	m0 245	−0.14	0.24	−0.13	0.29	−0.17	0.26	−0.26	0.17	−0.09	0.36	−0.11	0.24	−0.37	0.10	−0.07	0.27	−0.11	0.18
	m1 3																		
histological_type (Kruskal–Wallis test)	fibrolamellar carcinoma 2	1.65	0.44	2.51	0.29	2.2	0.33	2.89	0.24	2.76	0.25	3.94	0.14	2.23	0.33	3.28	0.19	2.40	0.30
	hepatocellular carcinoma 336																		
	hepatocholang-iocarcinoma 5																		
radiation_therapy (Wilcox test)	no 323	−0.07	0.59	0.02	0.80	0.03	0.98	−0.15	0.25	−0.01	0.54	−0.06	0.47	−0.14	0.24	−0.06	0.39	0.00	0.92
	yes 9																		
residual_tumor (Kruskal-Wallis test)	r0 303	1.58	0.45	0.96	0.62	2.04	0.36	2.64	0.27	1.77	0.41	1.59	0.45	1.83	0.40	2.46	0.29	1.02	0.60
	r1 15																		
	r2 1																		
ethnicity (Wilcox test)	hispanic or latino 17	−0.09	0.11	−0.01	0.37	−0.11	0.24	−0.09	0.25	−0.09	0.37	−0.04	0.41	−0.13	0.28	−0.10	0.49	−0.05	0.48
	not hispanic or latino 310																		

## Data Availability

The data used to support the findings of this study are included within the article.
